# Patient-derived stem cells: pathways to drug discovery for brain diseases

**DOI:** 10.3389/fncel.2013.00029

**Published:** 2013-03-27

**Authors:** Alan Mackay-Sim

**Affiliations:** National Centre for Adult Stem Cell Research, Eskitis Institute for Cell and Molecular Therapies, Griffith UniversityBrisbane, QLD, Australia

**Keywords:** embryonic stem cells, induced pluripotent stem cells, olfactory stem cells, olfactory neurosphere-derived cells, high content screening

## Abstract

The concept of drug discovery through stem cell biology is based on technological developments whose genesis is now coincident. The first is automated cell microscopy with concurrent advances in image acquisition and analysis, known as high content screening (HCS). The second is patient-derived stem cells for modeling the cell biology of brain diseases. HCS has developed from the requirements of the pharmaceutical industry for high throughput assays to screen thousands of chemical compounds in the search for new drugs. HCS combines new fluorescent probes with automated microscopy and computational power to quantify the effects of compounds on cell functions. Stem cell biology has advanced greatly since the discovery of genetic reprograming of somatic cells into induced pluripotent stem cells (iPSCs). There is now a rush of papers describing their generation from patients with various diseases of the nervous system. Although the majority of these have been genetic diseases, iPSCs have been generated from patients with complex diseases (schizophrenia and sporadic Parkinson’s disease). Some genetic diseases are also modeled in embryonic stem cells (ESCs) generated from blastocysts rejected during *in vitro* fertilization. Neural stem cells have been isolated from post-mortem brain of Alzheimer’s patients and neural stem cells generated from biopsies of the olfactory organ of patients is another approach. These “olfactory neurosphere-derived” cells demonstrate robust disease-specific phenotypes in patients with schizophrenia and Parkinson’s disease. HCS is already in use to find small molecules for the generation and differentiation of ESCs and iPSCs. The challenges for using stem cells for drug discovery are to develop robust stem cell culture methods that meet the rigorous requirements for repeatable, consistent quantities of defined cell types at the industrial scale necessary for HCS.

## INTRODUCTION

The last decades have seen development and applications in cellular neuroscience of DNA, RNA, and protein analysis technologies that provide large volumes of information, getting away from the traditional methods that follow the activities of single genes or single cell types. The application of DNA sequencing, RNA microarrays and higher throughput protein mass spectrometry is allowing examination of biological systems in all their complexity. What has been missing for cell biologists is the ability to interrogate cell functions at the same scale and level of complexity. This is being addressed by the developments in automated technologies developed by pharmaceutical companies to screen massive compound libraries for the discovery of new drug leads. Until recently this high throughput screening, with robotic control and automated data capture and analysis of experiments in 96-, 384-, and 1,536-well plates, centered on enzyme and receptor assays but increasingly, interest is turning to cell-based assays that capture the complexity of the environment in which drugs will actually operate. There has been a move to drug screening based on cellular outcomes (e.g., cancer cell apoptosis, inhibition of growth) rather than predicted mechanism (e.g., enzyme inhibition, receptor antagonism). This led to the concept and development of “high content screening” (HCS) that combines advances in fluorescence labeling of cells, robotic and automated microscopy, and automated image analysis that brings the analysis of cell functions to the high throughput formats of multiwell plates (Zanella et al., 2010). For the neuroscientist this technology opens up new frontiers in the ability to manipulate many experimental variables simultaneously in highly controlled experiments. For example, the effects of multiple drugs at multiple doses could be tested simultaneously on several cell types. With the many fluorescent reporter methods now available it is possible to follow three or four cellular events in the same experiment in hundreds of cells per well. With many well established platforms available, HCS opens the door to neuroscience for large scale, high throughput cell function analyses for understanding cell functions in health and disease.

In neuroscience, the discovery of the genetic causes of familial diseases has driven understanding of the functions of individual genes and proteins in cell function in the nervous system and the effects of mutations on brain function. Identification of a candidate gene is followed by genetically modified cell and mouse models to identify the functions of the identified gene at cell and systems level. Mouse models have been important for elucidating protein and gene functions but they often do not recapitulate human disease because the mice lack the human genetic background and the introduced human genes are acting in a non-human cellular context. Immortalized human cell lines have a more relevant genetic background but, being derived from tumors, they may not reflect a normal cellular context. Patient-derived primary cells might be a solution for both drawbacks but they cannot usually be maintained for very long in culture and finding an accessible cell type for brain diseases is problematic. Stem cell technologies may have the solution to these drawbacks.

There is an emerging interest in using stem cells to understand the cellular bases of human diseases. There is an imperative here, especially for neurological diseases and conditions. Large pharmaceutical companies are withdrawing from investing in neuroscience research because of the failure of the current paradigms to convert findings in animal models to drugs for human disease (Schnabel, 2008). There are obviously many reasons why animal models, genetic or otherwise, are not proving useful for predicting human responses to drugs. On the other hand, there are good reasons to expect that human stem cells might be useful, if they can be derived from patients with a disease and, if they are the cell types that are affected by the disease. This is clearly a niche that stem cells have the developmental abilities to occupy. Patient-derived stem cells could be used to identify cell functions that are altered by disease and thereby provide a target for drug discovery. Assays can then be developed for HCS to use the patient-derived stem cells to screen large drug libraries for therapeutic activity.

## HIGH THROUGHPUT SCREENING

**FIGURE 1:**
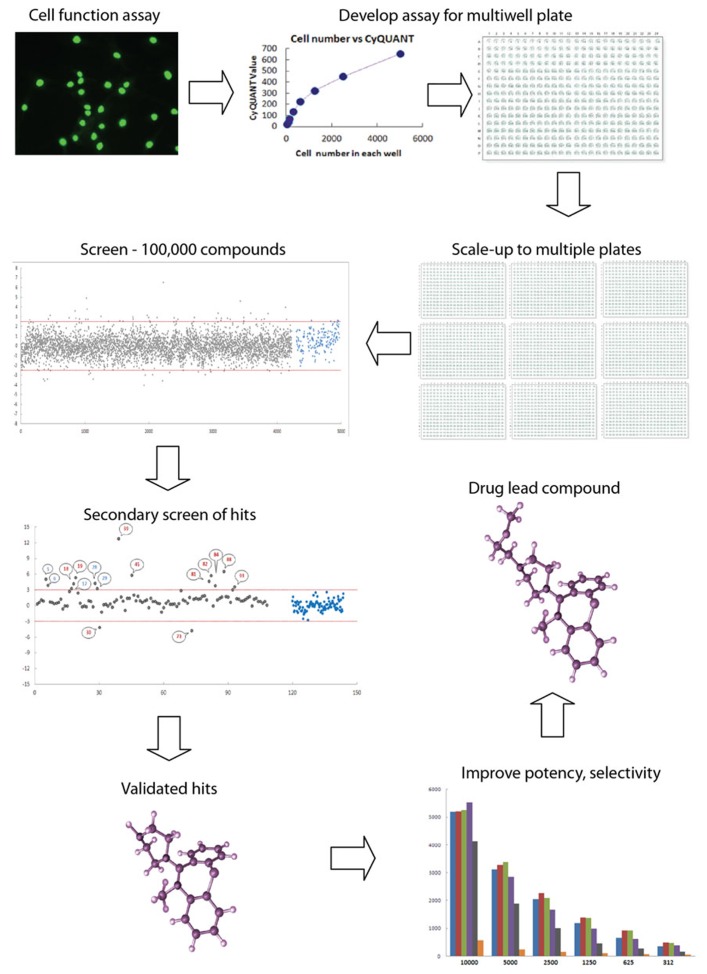
**High throughput screening: industrial scale biology.** High throughput screening is an automated process for conducting thousands of assays to identify active molecules modulating a biomolecular activity. Assays are performed in multiwell plates with robotics for cell and liquid handling and other aspects such as moving plates from incubator to drug delivery to plate reader. An assay for a cell function of interest must be validated for multiwell plate readers, such as fluorescence, absorbance, or luminescence. Here the assay is a fluorescence assay (Cyquant) for cell number. High levels of quality assurance and quality control are required to reduce the variance of the measurement so that the assay can be reliably scaled to multiple plates. Typically, thousands of compounds are screened for activity, with “hits” being defined as those compounds whose activity on the assay falls outside three standard deviations (red lines) around the mean of the controls on the plate (blue dots). Hits are the gray dots above and below the red lines. Secondary screens are then used to validate the initial hits. These screens may include dose–response curves or assays of related functions of interest, resulting in a selection of “validated hits,” which may comprise fewer than 1% of a typical library of compounds of varied structure. Here the validated hits are the numbered dots above the red line. Validated hits are then further assayed and modified chemically in an iterative process to improve potency, selectivity, pharmacological properties, and toxicological profile to produce a drug lead compound that will go into “preclinicalys” testing in animal models. High throughput screening is applied to large libraries of any potentially bioactive molecules including natural products, small molecule drugs, RNAs, and DNAs.

Increasingly in the last 20 years pharmaceutical companies have developed high throughput technologies to screen large chemical libraries of natural products and synthetic compounds for activities against selected enzymes and receptors, candidate targets for diseases of interest (Macarron et al., 2011; **Figure 1**). These high throughput technologies arose from the desire of pharmaceutical companies to test all theoretical chemical compounds (~100 million) against all theoretical human biological targets, estimated from the number genes (~20,000) or proteins (~200,000), indicating that as many as 10^12^ assays would be needed to identify all interactions between chemicals and targets (Sundberg, 2000). These technologies are pharmacology on a large scale, initially using 96-well plates and now routinely 384- and 1,536-well plates. The imperative is to provide a means to screen very large compound libraries in order to find the very small percentage of lead compounds that are active in a selected assay. High throughput screening is historically based on solution based enzyme and receptor assays using scintillation, absorbance, fluorescence, and chemiluminescence with the aim of finding highly specific chemical interactions with individual biological targets (Sundberg, 2000). High throughput screening is also used in cell-based assays, typically to monitor activation of cell surface receptors through subsequent transduction pathways, transcription events, cell proliferation, or cell death. These assays use multiwell plates and monitor colorimetric, fluorescence, luminance, or absorbance within each well. With developments in genetic technologies and the understanding provided by the Human Genome Project, intracellular events are available for high throughput screening through the development genetic fluorescent reporter assays. Cell lines have genes for luminescent or fluorescent proteins, like luciferase or green fluorescent protein (GFP), spliced into reporter systems to read out activation or inhibition of specific genes or proteins of interest (Sundberg, 2000). High throughput technologies have advanced the development of robotic automation for cell culture, assays, and compound library storage; automated and multipurpose plate readers; fluorescence dyes and reporter systems; computational power, and automated data storage and analysis. It currently takes about 1 week to screen 10,000 compounds against a target and 1–3 months to screen a library of one million compounds (Macarron et al., 2011). High throughput screening has been successful in delivering numerous drugs from discovery through to clinical use and the market starting from chemical libraries of 200,000–500,000 compounds (Macarron et al., 2011).

## HIGH CONTENT SCREENING

**FIGURE 2:**
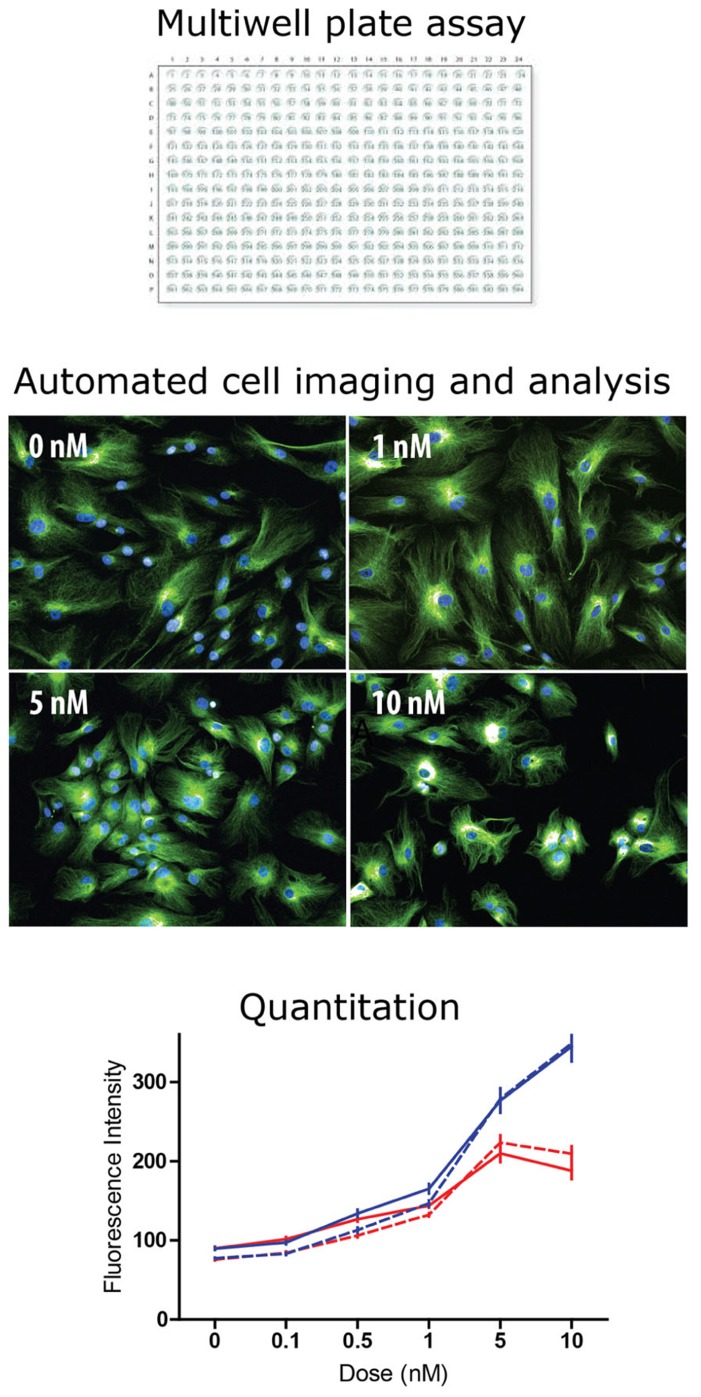
**High content screening: industrial scale microscopy.** High content screening is the automated microscopic analysis of fluorescence in cells of a multiwell plate. Most high throughput screening assays read activity per well (e.g., fluorescence, absorbance, and luminescence). In high content screening fluorescence in individual cells is quantified with automated image analysis. Typically, algorithms find the cells in the image and then measure fluorescence intensity and spatial distribution. The example shows tubulin fluorescence in cells (green) and cell nuclei (blue) at different concentrations of taxol, a tubulin-binding drug. The drug effect is difficult to see by eye but the analysis algorithm identifies each cell and quantifies tubulin fluorescence intensity, plotted here as a dose–response curve to two drugs, taxol (blue) and vinblastine (red) in two cell types: control-derived ONS cells (solid lines) and HSP patient-derived ONS cells (dashed lines; Abrahamsen et al., 2013). With high content screening thousands of cells can be analyzed to reveal small but significant differences between drugs, doses, and cell types.

High content screening is a further development in which the principles of high throughput screening are applied to the analysis of individual cells through the use of automated microscopy and image analysis (Zanella et al., 2010; **Figure 2**). This allows quantitative analyses of components of cells such as spatio-temporal distributions of individual proteins, cytoskeletal structures, vesicles, and organelles when challenged with chemical compounds. HCS can be used to monitor activation or inhibition of individual proteins and protein–protein interactions as well as allowing analysis of broader changes in biological processes and cell functions. Recent advances in the range of fluorescent probes for biological processes, functions, and cell components have combined with developments in fluorescence microscopy to give the cell biologists many new ways to understand cell functions in health and disease. These cell-centric developments have converged with high throughput concepts and with developments in automated microscopy and image analysis to evolve into the new technological synthesis of high throughput screening in which cell-based assays are conducted in multiwell plate formats. High content technologies are now used to screen chemical libraries for drug discovery as well as genome-wide RNA interference libraries to probe gene functions (Zanella et al., 2010). One of the advantages of HCS is the ability to apply different criteria to selected cells in the population to account for the heterogeneity of the cell population. For example, a chemical compound of interfering RNA may only act on cells in a particular stage of the cell cycle. Well-based assays provide a readout from the whole cell population, a mixture of cells in different phases of the cell cycle, whereas with cell-based HCS and appropriate markers, cells at identified phases of the cell cycle could be assessed independently. Thus HCS allows quantitative analysis of complex and heterogeneous cell cultures containing multiple cell types. Another advantage of HCS technologies is the ability to make multiple independent and quantitative measurements from single cells of interest. For example, transmitted light might be used to assess morphology; three or more fluorophores might be used to identify molecular components, structures, or organelles; and image analysis might be used to quantify spatial relationships between the fluorescent reporters under control conditions or when challenged with chemical compounds. With assays performed in multiwell plates there is opportunity to scale up experiments to include replicates, concentration–response curves, and parallel assessments of different cells or compounds. Modern instruments equipped with incubators and confocal optics have the ability to investigate cells over time in three spatial dimensions. These technologies will advance further and become cheaper allowing HCS principles to be applied increasingly by academic labs and not restricted to large pharmaceutical companies.

In the neurosciences, there is now an opportunity and challenge to combine patient-derived, disease-specific stem cells with HCS technologies with the aim of finding new drugs for brain diseases and conditions. This is not a simple aspiration because the majority of brain diseases are a result of complex genetic and environmental risk factors. Furthermore, brain diseases are usually just that, “brain” diseases and not “cell” diseases in the sense that cancers are. Nonetheless, it is possible that most brain diseases result from identifiable cellular dysfunctions such as those identified in monogenic disease. Such mutations tell us that specific brain dysfunctions can be manifest in specific cell types and pathways, despite universal genetic mutation. This gives hope that cellular models will shed light on the molecular and cellular mechanisms of emergent properties (e.g., cognition, emotion) evident when the brain functions as a whole.

## DISEASE-SPECIFIC PLURIPOTENT STEM CELL MODELS OF NEUROLOGICAL DISEASE

The analysis of gene function through gain- or loss-of-function in cell, fly, and mouse models has been very instructive in elucidating functions of genes and proteins but less successful in providing models that predict drug efficacy in human diseases. An example is the failure of the superoxide dismutase transgenic mouse model for amyotrophic lateral sclerosis that has yielded multiple compounds that are therapeutic in mouse but not humans (e.g., creatine; Klivenyi et al., 1999; Groeneveld et al., 2003). These problems arise because of species and model differences which do not mimic gene dosage, gene mutation variability or genetic background, or the complex of other contributing genetic factors that will be present in individuals with a disease. Patient-derived cells provide a potential solution to some of these problems because they reflect the genetic background and variation of the human population, arising from individuals with natural mutations. Sources of patient-derived cells have included easily accessible fibroblasts and lymphocytes and lymphoblastoid cell lines but there is concern that these cells will not manifest the same disease-associated properties as the cells specifically dysfunctional in the disease of interest, for example dopaminergic neurons of the substantia nigra in Parkinson’s disease or oligodendrocytes in multiple sclerosis. For such cell type-specificity the field holds high hopes of pluripotent stem cells which can theoretically be induced to differentiate into any cell type of interest.

Disease-specific pluripotent stem cells include human embryonic stem cells (ESCs) with genetic or chromosomal disorders derived from surplus blastocysts during *in vitro* fertilization and pre-implantation genetic diagnosis (Stefanova et al., 2012). Although these are not strictly “patient-derived” they carry specific genetic mutations or deletions that would normally lead to disease. Induced pluripotent stem cells (iPSCs) from patients have become the dominant choice for patient-derived pluripotent stem cells. A recent review lists 18 diseases for which ESCs have been derived, compared with 40 for which iPSCs have been derived (Grskovic et al., 2011). At this time many of the publications in this field are mainly demonstrations that pluripotent stem cells have been derived, often without demonstrating a disease-phenotype. Some show that the pluripotent cells can be differentiated into specific cell types of interest and some demonstrate deficits in cellular functions compared to control cells, as proof-of-principle for disease modeling (Grskovic et al., 2011; Maury et al., 2012). No doubt the numbers of diseases for which iPSCs are available will increase greatly in the next few years and deeper analyses of their functions will be forthcoming. It is a major challenge for the field to move beyond the proof-of-principle stage to discovery of new aspects of disease biology and new targets for therapeutic intervention.

The list of neurological diseases and conditions for which ESCs or iPSCs have been derived is largely limited to monogenic diseases including Charcot–Marie–Tooth disease type 1A, Down syndrome-trisomy 21, familial amyotrophic lateral sclerosis, familial dysautonomia, familial Parkinson’s disease, Fragile X syndrome, Friedreich ataxia, Gaucher’s disease, Huntington’s disease, Rett syndrome, Spinal muscular atrophy, spinocerebellar ataxia types 2 and 7, and X-linked adrenoleukodystrophy (Grskovic et al., 2011; Maury et al., 2012; Rajamohan et al., 2013). It is thought that diseases of complex genetics and environmental risk factors may be harder to model with pluripotent stem cells but patient-derived iPSCs have been generated from patients with Parkinson’s disease (Soldner et al., 2009) and schizophrenia (Brennand et al., 2011; Pedrosa et al., 2011). Patient-derived iPSCs from people with sporadic Parkinson’s disease were differentiated into dopaminergic neurons but failed to show an obvious difference in phenotype compared to control cells (Soldner et al., 2009). Similarly, a disease-associated phenotype could not be demonstrated in iPSCs from two cases of sporadic Alzheimer’s disease (Israel et al., 2012). In one study, iPSCs from schizophrenia patients were differentiated into neurons and gene expression profiling identified a cluster of differentially expressed genes involved in neurogenesis, neuronal differentiation, axon guidance, and adhesion with another cluster of differentially expressed genes involved in cell cycle regulation (Pedrosa et al., 2011). A second study in schizophrenia showed that neurons differentiated from patient-derived iPSCs had reduced neurite number and reduced connectivity with other neurons *in vitro* and reduced glutamate receptor expression (Brennand et al., 2011). These studies of patient-derived iPSCs from schizophrenia patients demonstrate that such models can reveal disease-associated cellular deficits in a disease of complex genetics, although the patients were all from families with psychosis, rather than sporadic cases.

It is a challenge to translate pluripotent cells into robust disease models (Maury et al., 2012). For example, ESCs are limited by the availability of genetic testing and pre-implantation genetic diagnosis. For their part, iPSCs are potentially compromised by the methods of their generation; most cell lines published to date were produced by integrating vectors, although this will change as the efficiency and predictability of non-integrating methods improves. There are other technical challenges for disease modeling such as the current lack of robust and efficient protocols for differentiating ESCs and iPSCs into disease-associated cells of interest. Attention is drawn to the importance of selecting appropriate matched controls because case–control cell differences could arise from sampling bias and “disease-associated” differences may result from particular comparator control cells (Zhu et al., 2011; Maury et al., 2012). This difficulty is compounded by the large cost of generation and maintenance of ESCs and iPSCs that will limit sample sizes for most laboratories. One preferred method would be to use each patient-derived cell line as its own control by correcting its genetic defect (Zhu et al., 2011; Maury et al., 2012) but this is possible only in the monogenic disease cases of known mutations that can be selectively corrected. It is expected that many of these challenges will be overcome as new methodologies develop for efficient production of patient-derived iPSCs that are generated with non-integrating reprograming methods (Grskovic et al., 2011) and efficient methods are devised for differentiation into desired cell types: specific neurons, astrocytes, and oligodendrocytes.

## PATIENT-DERIVED OLFACTORY STEM CELLS AS MODELS FOR NEUROLOGICAL DISEASES

Published studies of ESCs and iPSCs as disease models are all confined to small numbers of cell lines from patients and controls. This makes it difficult to generalize from these case–control studies to the general population. Variability in the reprograming process and epigenetic status makes it essential that several clones from several individuals are compared to confirm that a “disease-phenotype” is not confounded by individual differences among case or control cell lines (Vitale et al., 2012). Ideally, patient- and control-derived cells should be sampled from large populations to be certain that differences between the samples are representative of the population. In the case of genetic diseases with high penetrance of the clinical phenotype, the case–control design may be robust to small sample size but this may not be true of lower penetrance phenotypes, or of sporadic diseases of complex genetics. This sampling problem can be overcome with accessible cells that can be derived easily from larger numbers of individuals, such as patient-derived fibroblasts and lymphocytes, which have been used to identify potential cellular mechanisms or biomarkers of neurological diseases including Alzheimer’s disease (Takahashi et al., 1999; Moreira et al., 2007), Parkinson’s disease (Martin et al., 1996; Hoepken et al., 2008), and schizophrenia (Ilani et al., 2001; Wang et al., 2010). Although these cell types have identified cellular and molecular differences between patients and controls, their relevance to diseases of the nervous system is moot and the patient–control differences can be non-existent (Matigian et al., 2008).

Another approach to modeling diseases is to sample patient-derived adult stem cells. Neural progenitor cells were isolated from post-mortem brain from Alzheimer’s patients and healthy controls (Lovell et al., 2006). Mesenchymal stem cells were isolated from controls and patients with amyotrophic lateral sclerosis (Ferrero et al., 2008) and Parkinson’s disease (Zhang et al., 2008). None of these studies reported disease-associated differences, on the contrary all stated that patient and control cells were of similar phenotype.

There is a multipotent adult stem cell resident in the olfactory mucosa, the organ of smell in the nose (Murrell et al., 2005). A stem cell in the olfactory epithelium maintains a continual neurogenesis throughout life that regenerates the sensory neurons (Mackay-Sim and Kittel, 1991a,b; Murrell et al., 1996). Olfactory epithelium is one of the few places in the nervous system that contains a neural stem cell but, unlike the other sites in the brain, it is accessible under local anesthetic (Feron et al., 1998) and several studies have shown disease-associated differences in cell biology in this tissue. Olfactory neuroblasts isolated from post-mortem epithelial biopsies from controls and individuals with Alzheimer’s disease demonstrated differences in amyloid precursor protein processing (Wolozin et al., 1993) and oxidative stress (Ghanbari et al., 2004). Histological analysis of olfactory epithelium indicates disturbed neural differentiation in schizophrenia (Arnold et al., 2001) and Rett syndrome (Ronnett et al., 2003). Cultures of olfactory mucosa show increased cell proliferation and reduced tissue adhesion in schizophrenia compared to healthy controls (Feron et al., 1999; McCurdy et al., 2006). Clearly the olfactory organ demonstrates disease-associated differences in cell biology in several brain diseases including a monogenic disease (Rett syndrome) and complex genetic diseases of no known genetic cause (Alzheimer’s disease and schizophrenia). This led to the development of an olfactory stem cell-based system for investigating brain diseases based on olfactory neurosphere-derived cells (ONS cells; Matigian et al., 2010).

The olfactory mucosa comprises the superficial epithelium and the underlying lamina propria separated by a basement membrane. Within the epithelium are basal cells among which are the multipotent stem cells that can regenerate all the cell types of the epithelium including the sensory neurons as well as other non-neural supporting and gland cells (Leung et al., 2007; Packard et al., 2011). Within the human lamina propria is a multipotent stem cell with characteristics both of neural and mesenchymal stem cells, an ectomesenchymal stem cell (Delorme et al., 2010). The lineage relation between these stem cells, if any, is not known. When biopsies of human olfactory mucosa are dissociated and grown in a serum-free medium containing epidermal growth factor (EGF) and basic fibroblast growth factor 2 (FGF2), neurospheres form that can differentiate into neurons and glia and cells of non-ectodermal lineage, including developing cardiac and skeletal muscle, kidney and liver, and blood (Murrell et al., 2005). Neurospheres are dissociated and when the cells are grown as adherent cultures in a serum-containing medium, these “neurosphere-derived” cells (ONS cells) have a flattened, undifferentiated appearance with a marker phenotype similar to the ectomesenchymal cells derived from primary cultures of olfactory mucosa (Delorme et al., 2010; Matigian et al., 2010), that is, they express markers of both neural and mesenchymal lineages. Patient-derived ONS cells show robust disease-specific differences compared to ONS cells derived from healthy controls (Mackay-Sim, 2012). Gene expression profiling of ONS cells from healthy controls and patients with Parkinson’s disease or schizophrenia showed disease-specific differences in gene expression, protein expression, and cell functions (Matigian et al., 2010). Disease-associated gene expression differences were quite different in schizophrenia and Parkinson’s disease, with alterations in neurodevelopmental pathways in schizophrenia and in oxidative stress and metabolic function in Parkinson’s disease (Matigian et al., 2010). Deeper analysis of the gene expression showed disease-specific differences in the variance of gene expression in the major signaling pathways. Overall schizophrenia patient-derived ONS cells were less variant in their gene expression compared to controls whereas Parkinson’s patient-derived ONS cells were more variant than controls, indicating another dimension along which diseases can affect cell functions (Mar et al., 2011).

Parkinson’s patient-derived ONS cells showed gene expression and functional differences indicating dysfunctions in pathways involved mitochondrial metabolism and oxidative stress (Matigian et al., 2010). Pathway analysis of the gene expression differences found that the antioxidative nuclear factor (erythroid-derived 2)-like 2 (NRF2) signaling pathway was overrepresented among the dysregulated genes (Matigian et al., 2010). NRF2 is a transcription factor that ameliorates the effects of oxidative stress. Deeper analysis of the ONS cells demonstrated that the NRF2 signaling pathway was dysfunctional: although the NRF2 protein was equally expressed in patient and control cells the downstream targets of NRF2 were reduced, suggesting lesser activation of NRF2 signaling in the patient cells (Cook et al., 2011). In contrast, schizophrenia patient-derived ONS cells showed gene expression and functional differences indicating dysfunctions in pathways involved neurodevelopment (Matigian et al., 2010). Pathway analysis of the gene expression differences identified aspects of the cell cycle that were dysregulated, in particular G1/S phase transition, the check point in cells that controls the beginning of DNA synthesis (Matigian et al., 2010). Functional analysis demonstrated that patient-derived cells proliferated faster than control-derived cells in accord with previous observations in olfactory biopsy cultures (Feron et al., 1999; McCurdy et al., 2006). This faster rate of cell proliferation was due to a 2-h shorter cell cycle period in patient cells (Fan et al., 2012). G1/S phase transition is dependent on the intracellular levels of cyclin D1, a cell cycle control protein; cyclin D1 levels were elevated in patient cells and reached higher levels more quickly than control cells (Fan et al., 2012). In schizophrenia, ONS cells gene expression was significantly dysregulated in the focal adhesion kinase signaling pathway, which is involved in regulating attachment to the extracellular matrix through cell surface integrin receptors (Fan et al., 2013). Functional analyses of the patient-derived cells showed they were less adhesive and more motile than control-derived cells with smaller and fewer sites of adhesion that disassembled more quickly than in control-derived cells (Fan et al., 2013). These studies demonstrate that patient-derived ONS cells can show robust disease-specific differences in cell biology even in sporadic diseases of complex genetics.

Olfactory neurosphere-derived cells are also proving useful for understanding monogenic diseases. Hereditary spastic paraplegia (HSP) is an autosomal dominant disease affecting the long spinal axons from the motor cortex to the lower motor neurons in the spinal cord. ONS cells from patients with HSP were similar in many basic cell functions to ONS cells from healthy controls despite dysregulation of expression of 60% of the genome, indicating a high level of homeostatic regulation in response to dominant mutations in *SPAST*, which codes for a microtubule severing protein (Abrahamsen et al., 2013). Closer inspection of cell functions using HCS revealed significant reductions in stable microtubules and in the intracellular distributions of peroxisomes and mitochondria (Abrahamsen et al., 2013). Live-cell time-lapse imaging revealed that peroxisomes traveled more slowly in HSP patient-derived cells, consistent with the corticospinal axon pathology in HSP (Abrahamsen et al., 2013). Ataxia telangiectasia (AT) is a fatal autosomal recessive disease characterized by radiation sensitivity, cancer, and cerebellar dysfunction. ONS cells from children with AT had radiation sensitivity and DNA-repair deficits that were corrected by insertion of the full-length gene (Stewart et al., 2013). Immature neurons from AT patient-derived ONS cells showed evidence of impaired differentiation (Stewart et al., 2013). Olfactory ectomesenchymal stem cells, similar to ONS cells, demonstrated a disease-associated effect of mis-splicing of the *IKBKAP* gene leading to reduced levels of IKBKAP protein and altered cell migration in patient-derived cells in familial dysautonomia (Boone et al., 2010).

## PATIENT-DERIVED OLFACTORY STEM CELLS FOR DRUG DISCOVERY

Patient-derived ONS cells have several advantages for HCS for drug lead identification. They are cheap to grow and maintain, growing in standard cell culture conditions with no expensive growth factors after the neurosphere-forming stage. As ONS cells they can be grown for at least 16 passages without significant change in gene expression thus demonstrating minimal phenotypic change and without change in karyotype (unpublished observations). ONS cells are derived from neural tissue and can obviously show disease-specific phenotypes relevant to the neurological diseases from which the donors suffer. Proof-of-principle analyses have shown that brain diseases “ain a dish” can be ameliorated by drug treatment. For example, Parkinson’s patient-derived ONS cell functions were restored to control-derived cell levels by treatment with L-sulforaphane, an agonist of NRF2 (Cook et al., 2011). Similarly, a small molecule drug kinetin was able to reverse the mis-splicing of the *IKBKAP* gene in familial dysautonomia patient-derived ectomesenchymal cells (Boone et al., 2010) as it has in iPSCs from these patients (Lee et al., 2009). HCS was used to test the differential sensitivity of HSP patient-derived and control-derived ONS cells to the tubulin-binding drugs, taxol and vinblastine (Abrahamsen et al., 2013). HSP patient-derived cells had 50% the control level of spastin, a tubulin-severing enzyme, 150% of the control level of stathmin, a tubulin depolymerizing enzyme, and 50% of the control level of acetylated a-tubulin, an indicator of stabilized microtubules (Abrahamsen et al., 2013). HCS showed that patient-derived and control-derived ONS cells were highly sensitive to the drugs, with effects at 0.1 nM (**Figure 2**). Both drugs increased acetylated a-tubulin, but with different dose–response curves, and low doses of both drugs (~0.3 nM) restored patient cell acetylated a-tubulin to the control cell level (Abrahamsen et al., 2013).

These experiments show that disease-associated dysfunctions in olfactory cells can be ameliorated by candidate chemical compounds acting on targets known to be disrupted in the patient-derived cells compared to controls. The next challenge is to see whether ONS cells are useful for screening libraries of compounds. They have some of the necessary characteristics such as ease of generation, low cost, robust and repeatable growth characteristics and predictable phenotype. These properties make them useful for building up banks of cells that will allow assessment of variability of cell biology across a wider population of patients and controls, to discriminate disease-specific differences from individual differences in complex diseases like Parkinson’s disease and schizophrenia.

## FUTURE PROSPECTS

One of the challenges for the field is to develop robust and repeatable protocols for producing the large quantities of specified neurons or glia that are required for high throughput screening. For ESCs and iPSCs differentiating protocols exist for making different types of neurons, such as dopaminergic neurons, cortical neurons, and motor neurons (Chambers et al., 2009) but the yield is generally low and variable. This is not necessarily limiting for low-throughput investigations of disease-associated cell phenotypes and lead validation but is certainly limiting for primary drug screens. ONS cell production is not limiting for drug discovery but they currently also lack robust and repeatable protocols for differentiating them on demand into neurons and glia *in vitro*. In growth factor-free medium 50% of the cells were “astrocytes” [glial fibrillary acidic protein (GFAP)-positive cells], a proportion elevated by ciliary neurotrophic factor (Murrell et al., 2005). “Neurons”(β-tubulin-III-positive cells) were less frequent but rose to 25% of the population with nerve growth factor in the medium. (Murrell et al., 2005). Retinoic acid induced a majority (50%) of O4-positive “oligodendrocytes” (Murrell et al., 2005). ONS cell differentiation was defined in terms of morphology and immunofluorescence and so lacks the definitive demonstration of differentiation shown repeatedly for ESCs and IPSCs.

**FIGURE 3:**
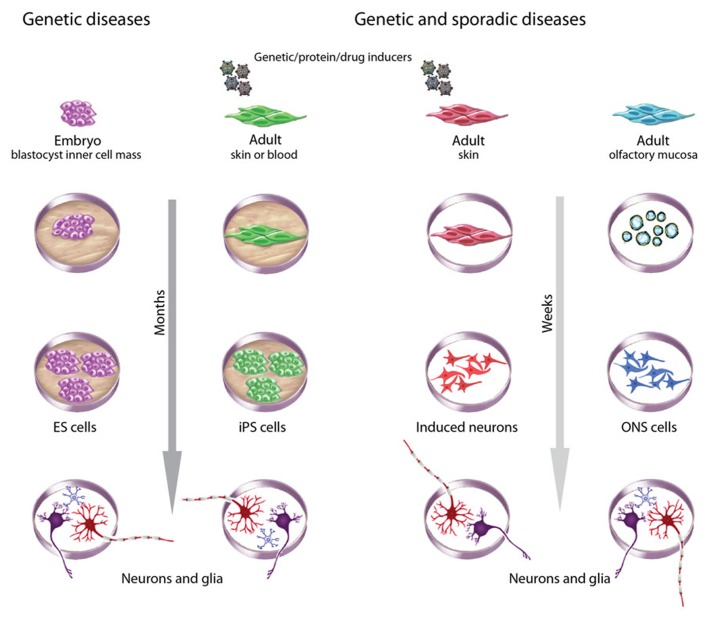
**Choices of stem cells to model brain diseases.** Different criteria guide choices of cells to model brain diseases. ESCs derived during pre-implantation genetic diagnosis are useful for monogenic diseases. Patient-derived adult cells are useful for genetic and sporadic diseases, with the advantage of an associated clinical history. ESCs and iPSCs take many months to generate, validate, and then to produce neurons and glia but have the advantage of being highly proliferative and pluripotent. iPSCs and induced neurons require reprograming with genes, proteins or drugs, whereas ESCs and ONS cells do not. ONS cells and induced neurons may retain the methylation status of differentiated cells whereas ESCs and iPSCs do not. All methods introduce variability associated with cell culture but iPSCs and induced neurons may be more variable because of clonal selection due to the low efficiencies of the induction processes. ESC and ONS cell production average inter-clonal variation across large populations.

The concept of drug discovery through patient-derived stem cell models of brain diseases is attractive but has many other challenges apart from the practical issues of cost and reliable production. Concerns are raised about the epigenetic status of iPSCs and ESCs – epigenetic status is variably altered by reprograming and by culture methods (Stadtfeld and Hochedlinger, 2010) hence diseases in which epigenetics plays a role may not be modeled well by iPSCs and ESCs (Zhu et al., 2011; Maury et al., 2012). There is not yet a consensus about which cell type is the best for reprograming (Stadtfeld and Hochedlinger, 2010) or which method of reprograming is the most robust and reliable (Grskovic et al., 2011). Differentiation of human iPSCs is not yet routine and predictable and can take many weeks (Grskovic et al., 2011). There is discussion about the applicability of iPSCs to model late-onset diseases like Parkinson’s disease or Alzheimer’s disease, with the inference that iPSCs may be better suited to neurodevelopmental disease modeling (Juopperi et al., 2011). Olfactory stem cell models appear to be suited to late-onset and sporadic diseases perhaps because they are not subject to the genetic and epigenetic changes due to reprograming. Olfactory stem cells are relatively inexpensive and can be reliably grown in large quantities but they also suffer from difficulties in robust and routine differentiation protocols. As more diseases get modeled by iPSCs and ONS cells the strengths and weaknesses of each model will be determined for specific applications and the relevance of each model to the disease of interest will be determined. The use of particular cell models for drug discovery will depend on many variables including relevance to the disease, ease and cost of use, and stage of the drug discovery process (**Figure 3**). These questions and challenges are all signs of a field at the very beginning of its genesis and many will undoubtedly be resolved in the coming years.

Other developments in reprograming will affect this future. It is now possible to generate neurons directly from skin fibroblasts (Vierbuchen et al., 2010). This may greatly reduce the cost of producing patient-derived neurons for drug discovery because fibroblasts, like ONS cells are cheaper to generate, expand, and maintain than iPSCs. Through similar direct reprograming it may be possible to on demand specific classes of neurons, astrocytes, and oligodendrocytes. Ultimately whether any of these new technologies reach routine application in drug discovery will depend on cost, robustness, and industrial scalability as well as the biological validity of the cells as disease models.

## Conflict of Interest Statement

The author declares that the research was conducted in the absence of any commercial or financial relationships that could be construed as a potential conflict of interest.
